# Evaluating Sensor Fusion and Flight Parameters for Enhanced Plant Height Measurement in Dry Peas

**DOI:** 10.3390/s25082436

**Published:** 2025-04-12

**Authors:** Aliasghar Bazrafkan, Hannah Worral, Cristhian Perdigon, Peter G. Oduor, Nonoy Bandillo, Paulo Flores

**Affiliations:** 1 Department of Agricultural and Biosystems Engineering, North Dakota State University, Fargo, ND 58102, USA; aliasghar.bazrafkan@ndsu.edu (A.B.);; 2 Department of Plant Science, North Dakota State University, Fargo, ND 58102, USA; 3 Department of Landscape Architecture, Disaster Resilience, and Emergency Management, North Dakota State University, Fargo, ND 58102, USA; 4 Department of Crop and Soil Sciences, North Carolina State University, Raleigh, NC 27606, USA

**Keywords:** digital aerial photogrammetry, high-throughput phenotyping, LiDAR, plant height, UAS

## Abstract

Plant height is an important trait for evaluating plant lodging, drought, and stress. Standard measurement techniques are expensive, laborious, and error-prone. Although UAS-based sensors and digital aerial photogrammetry have been tested on plants with an erect growth habit, further study is needed in the application of these technologies to prostrate crops such as dry peas. This study has compared the performance of LiDAR, RGB, and multispectral sensors across different flight configurations (altitudes, speeds), and image overlaps over dry pea plots to identify the optimal setup for accurate plant height estimation. Data were assessed to determine the effect of sensor fusion on plant height accuracy using LiDAR’s digital terrain model (DTM) as the base layer, and digital surface models (DSMs) generated from RGB and multispectral sensors. All sensors, particularly RGB, tended to underestimate plant height at higher flight altitudes. However, RMSE and MAE values showed no significant difference, indicating that higher flight altitudes can reduce data collection time and cost without sacrificing accuracy. Multispectral and LiDAR sensors were more sensitive to changes in flight speed than RGB sensors; However, RMSE and MAE values did not vary significantly across the tested speeds. Increased image overlap resulted in improved accuracy across all sensors. The Wilcoxon–Mann–Whitney test showed no significant difference between sensor fusion and individual sensors. Although LiDAR provided the highest accuracy of dry peas height estimation, it was not consistent across all canopy structures. Therefore, future research should focus on the integrating machine learning models with LiDAR to improve plant height estimation in dry peas.

## 1. Introduction

Pulse crops such as dry peas, chickpeas, and lentils are important for improving nutrition [1], sustainable food production [2], economic development [3] and public health [4]. They are also integral to global food systems and contribute significantly to the preservation of environmental sustainability. Measuring the height of pulse crops is essential for monitoring plant growth [5], predicting yield [6] conducting phenotypic analysis [7] and assessing environmental impact [8]. These factors are central to sustainable agriculture and plant science research.

Currently, measuring plant height is a time-consuming and manual process, often prone to error. In contrast, remote sensing offers a more precise and less labor-intensive alternative. Remote sensing technologies such as Light Detection and Ranging (LiDAR) and photogrammetry, can collect detailed and accurate plant height data across large areas. These methods operate in a fraction of the time required by traditional methods. These technologies can be deployed from uncrewed aerial systems (UAS), satellites, or surface platforms, enabling detailed, long-term commercial monitoring of plant growth.

LiDAR is a system that emits laser pulses toward the ground [8]. When these pulses strike an object, like a plant, some of the light is reflected to the sensor, which measures the time it takes for the pulse to return (“time of flight”) [9]. The distance to the object is then calculated based on this time. As the pulse travels through vegetation, it reflects off multiple surfaces (e.g., canopy, branches, ground), resulting in several returns. The first return typically comes from the top of the vegetation, while the last return is usually from the ground. The system generates a dense “point cloud” from millions of these returns. By analyzing this point cloud, the height difference between the ground surface and the canopy elevation can be determined, providing the final plant height measurement.

In photogrammetric measurement of plant height, the acquisition of multiple overlapping images covering the area of interest is followed by a sequence of processing steps which allow the creation of a three-dimensional model of both the terrain and the vegetation [10]. Photogrammetry is based on a technique called Structure from Motion (SfM) [11], which compares the images to identify common points across them [12]. It then reconstructs the 3D structure of the scene by determining the relative positions of these points. Similarly to LiDAR, photogrammetry creates a point cloud, but in this case, the points are derived from the 2D images rather than laser reflections. This point cloud consists of millions of points, and each is represented by X, Y, and Z coordinates that map the surfaces of the ground, buildings, and vegetation.

While the capabilities of LiDAR and photogrammetry for estimating plant height have been demonstrated for numerous crops, most previous UAS remote sensing studies have dealt with relatively erect crops, e.g., wheat [13,14] and corn [15,16,17], creating a research gap in developing accurate plant height metrics for low-stature crops like the dry pea. Low-lying crops have unique structural characteristics that make it challenging to extract reliable plant height information from LiDAR, RGB, and multispectral imagery. The literature still lacks detailed evaluations of specific sensor performance under varying flight configurations, as well as assessments of the potential benefits of sensor fusion. Moreover, while LiDAR can provide high accuracy with tall crops, its effectiveness in estimating the height of short, densely canopied crops is less documented, indicating that more research should be conducted on the reliability of short crops.

This study aims to address existing gaps in the use of UAS-based remote sensing technologies for estimating the height of short-stature plants, particularly the dry pea. Specifically, we explore the optimization potential of different flight configurations by comparing the height estimation capabilities of LiDAR, RGB, and multispectral sensors across various flight configurations such as altitudes, speeds, and image overlaps. In addition, this research evaluates the effect of sensor fusion to overcome known limitations, especially the underperformance of LiDAR in capturing complex, low-lying canopy structures.

## 2. Materials and Methods

**Figure 1:**
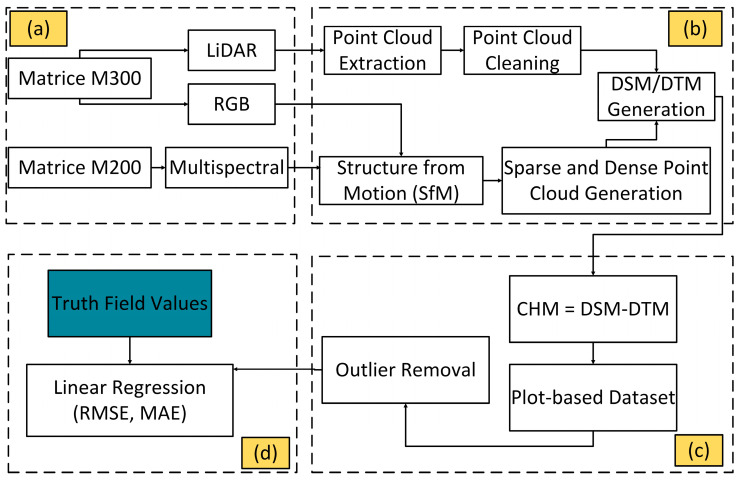
Workflow for estimating plant height in dry peas using different UAS sensors includes: (**a**) image acquisition; (**b**) image processing; (**c**) dataset creation and cleaning; and (**d**) model evaluation. CHM—canopy height model; DSM—digital surface model; DTM–digital terrain model.

The goal of this study was to evaluate the performance of different sensor types and flight patterns for estimating dry pea plant height to form a complete data processing workflow (Figure 1), from the acquisition of raw sensor data (step a), production of point clouds and height models (step b), creation of a cleaned plot-level dataset (step c), and accuracy assessment carried out by end users (step d).

### 2.1. Field Trial

The experiment was conducted during the 2023 crop season at North Dakota State University’s North-Central Research Extension Center in Minot, ND. The field trial was set out with 307 plots (9.0 × 1.5 m), including 204 unique dry pea genotypes, sown on May 5th and harvested on August 8th. We measured plant height (m) at two randomly selected sites at opposite ends of each plot. We used a measuring stick marked in 1 mm increments to measure the distance of the plant’s apical meristem with a maximum measurement error of ±1 mm in height measurement. In cases where plants were inaccessible or damaged, the height was estimated by averaging the height of the closest plants immediately adjacent. We then computed the average height value for all these measurements for each plot. The plot-level mean heights served as the ground truth data, which served as the ground truth data for comparison between our remote sensing estimates and actual plant height. Due to the small stature of dry pea plants, a measuring stick with an accuracy of ±1 mm was found to capture height variability at a fine scale while providing high-confidence measurements. Since this study was performed on the plot scale, a team assigned from the plant breeding group needed to evaluate the field in the growing season to verify the identity of the plants and minimize interference from non-target vegetation. The team kept track of plant growth and removed undesirable plants that would affect remote sensing measurements to ensure the consistent integrity of the experimental plots. Specifically, vegetation not included in the experimental treatment and located within the plots between in situ data collection points was mowed before each round of data collection. This was performed to ensure that height measurements derived from remote sensors were not affected by weeds, other plants, or ground-level obstructions. Similarly, only the targeted dry pea plants were maintained at the same height to enable analysis of height. Using this technique, dry peas could be accurately separated from neighboring objects without any need for additional image segmentation approaches. Field evaluation was conducted at the same time each day to ensure a similar illumination and reduce variability based on the variation in light. By eliminating extraneous vegetation in the inter-plot areas and ensuring controlled field conditions, we minimized sources of error in plant height estimation and improved the reliability of sensor-based measurements.

### 2.2. Image Acquisition

To assess the UAS data, different sensors were used, including the Zenmuse L1 (DJI Inc. in Shenzhen, China) for LiDAR, the Zenmuse P1 (DJI Inc. in Shenzhen, China) and Sentera 65r (Sentera, Minneapolis, MN, USA) for RGB, and the RedEdge-MX dual camera system (AgEagle, Wichita, KS, USA) for multispectral data. The DJI Matrice 300 aircraft (DJI Inc., Shenzhen, China) was used to fly the LiDAR and RGB sensors, while the DJI Matrice 200 (DJI Inc., Shenzhen, China) was used for the multispectral sensor. DJI Pilot version 2.5.1.15 (DJI Inc., Shenzhen, China) was used for aerial operations with both UAS. All the UAS flight experiments were conducted in the same environmental conditions, between 10:00 AM and 2:00 PM, to minimize the effects of diurnal variations in illumination. Flights were carried out on days with calm weather and low wind speeds (<5 mph), and data were collected under clear skies or uniformly overcast conditions, which provided homogeneous illumination over the field.

### 2.3. Image Processing

For the plot-level analysis, we employed a semi-automated technique using ArcGIS Pro version 3.0.3 (Esri, Redlands, CA, USA) to delineate the boundaries of each plot. This was accomplished by utilizing the RGB orthomosaic from the Sentera 65r (higher spatial resolution). By inputting the coordinates of the lower-left corner of the plots along with their dimensions and the distances between them, both vertically and horizontally, the tool was able to automatically define the plot boundaries. Each plot was then assigned a unique ID, corresponding to the ID used for field data collection, to ensure accurate matching of remote sensing data with the in situ measurements. LiDAR data were processed using DJI Terra version 3.5.5 (DJI Inc., Shenzhen, China) to create a dense cloud layer. Subsequently, ArcGIS Pro was used to generate a digital surface model (DSM) and a digital terrain model (DTM) from the point cloud layer. Regarding the RGB and multispectral sensors, Pix4DMapper from Pix4D (Pix4D, Lausanne, Switzerland) version 4.8.1 was used to generate the DTM and DSM layers. The DSM represents all features above the ground, while the DTM indicates the elevation of the bare ground. Finally, a Structure from Motion (SfM) approach [18] was applied within ArcGIS Pro version 3.0.3 to derive the plant height layer by subtracting the DTM from the DSM.

### 2.4. Data Analyses

#### 2.4.1. Dataset Creation and Cleaning

After extracting the boundary of each plot and obtaining the DSM and DTM, a script was developed in ArcGIS Pro version 3.0.3 to calculate the minimum (min), mean, median, maximum (max), standard deviation (stdev), and percentiles (5th, 10th, 25th, 50th, 70th, 80th, 90th, and 95th) values of plant height for each plot based on the subtraction of DTM values from DSM values. This script follows a zonal statistic approach to calculate these metrics for each, which are then saved into an Excel table, creating the initial dataset for each flight configuration. For data quality control, the Shapiro–Wilk [19] and Kolmogorov–Smirnov (KS) tests [20] were conducted on the datasets. The Shapiro–Wilk test is a statistical test that checks whether a sample comes from a normally distributed population. For data quality control, we used the Shapiro–Wilk [20] and Kolmogorov–Smirnov (KS) [21] tests to assess the normality of the datasets. While the Chi-square test is another method used for normality testing, it is generally more sensitive to binning strategies and is less powerful than the Shapiro–Wilk test for continuous, unbinned data, especially when accurate interval grouping is not available [21]. The Shapiro–Wilk test, despite being originally developed for smaller sample sizes, is known to be more robust and powerful for detecting departures from normality in continuous data, even with moderately large samples (*n* < 2000) [22]. For these reasons, we selected the Shapiro–Wilk test along with the KS test, both of which are more appropriate for our unbinned, continuous datasets in this context. The KS test is a non-parametric test that compares the sample distribution with a reference probability distribution (normal distribution). If the *p*-value is less than a chosen significance level (0.05), we reject the null hypothesis and conclude that the data are not normally distributed. These tests indicated that while data from different flight altitudes conformed to a normal distribution, the datasets associated with varying speeds and overlaps did not. Instead of implementing transformations (e.g., Box–Cox method [23]) to the data, which can alter the data distribution and potentially bias the interpretation [24], the Wilcoxon–Mann–Whitney test [25], a robust non-parametric alternative, was employed. This test does not assume normality and is well suited for datasets with skewed distributions or outliers, ensuring that statistical comparisons remain valid and reflective of the true data characteristics [26]. To ensure data quality and reduce the influence of erroneous or extreme values, a Z-score method [27] was employed to detect and remove outliers. In the context of high-throughput phenotyping, such outliers are often attributed to factors like sensor noise, occlusion by surrounding vegetation, or irregular ground reflections that can distort canopy height estimation. A threshold of Z > 3 was chosen based on initial exploratory analysis, aligning with standard statistical conventions for identifying extreme values in normally distributed data. This threshold effectively balances sensitivity to abnormal values while minimizing the loss of valid observations. Approximately 8.8% of data points were identified and excluded as outliers. To ensure consistency, the same outlier detection procedure was applied uniformly across all datasets and sensor configurations. The resulting cleaned dataset comprised 280 plots and was used for all subsequent statistical and comparative analyses. This step was essential to reduce bias and improve the robustness of height estimation comparisons across sensors and flight parameters. Residual plots [28], which show the difference between actual values and predicted values (residuals) of plant height, were used to assess the suitability of different height metrics (e.g., maximum, mean, median, percentiles, standard deviation) under various flight configurations (including different altitudes, speeds, and overlaps). Since the UAS-based reconstruction of plant height produces high-density point clouds, direct use of raw point-level data is impractical for statistical comparison. Instead, these metrics were computed to summarize the height information per plot. Plotting the residuals (actual-predicted) helps to identify any patterns in the residuals and assess prediction accuracy [29]. A good fit will have residuals randomly scattered around zero with no apparent pattern. To evaluate whether any metric showed statistically significant differences in estimation accuracy, we initially considered a *t*-test [30]; however, Shapiro–Wilk and Kolmogorov–Smirnov tests revealed that the residuals for most metrics were not normally distributed. Therefore, we employed the Wilcoxon–Mann–Whitney test, a non-parametric alternative suitable for non-normally distributed data. This approach ensures a more robust and appropriate comparison of metric performance in plant height estimation for dry pea.

#### 2.4.2. The Effect of Flight Configurations on the Estimation of Plant Height

To assess the effect of different flight configurations on the estimation of plant height in dry pea, each sensor was flown at four flight altitudes (15, 30, 46, and 61 m (m) above ground level (AGL)), three flight speeds (5 m.s^−1^, 10 m.s^−1^, and 15 m.s^−1^), and four levels of side and forward image overlaps (50%, 60%, 75%, and 85%). Image overlap refers to the percentage of coverage that each image shares with adjacent images in the flight grid. When flying at different altitudes, the flight speed was set to 10 m.s^−1^ and image overlap was set to 75% for all sensors. When flying at different flight speeds, the flight altitude was set to 46 m, and image overlap was set to 75%. When flying with different image overlaps, flight altitude and speed were set to 46 m and 10 m.s^−1^, respectively. To show the impact of flight variables on the estimated value of plant height using different sensors, the variation in the estimated plant height values for the selected metric was plotted for different flight altitudes, speeds, and image overlap levels. Additionally, a scatter plot was used to show the predicted values of plant height versus the actual values of plant height under different flight settings for each sensor. These scatter plots illustrate how changes in flight configurations lead to underestimation, overestimation, or accurate estimation of plant height. The values of root mean square error (RMSE) [31], mean absolute error (MAE) [32], and the coefficient of determination (R^2^) [33] were used to compare the estimated values of plant height with the actual values. RMSE (Equation (1)) measures the average magnitude of the errors between the remote sensing estimates and the actual plant heights. MAE (Equation (2)) measures the average absolute errors between the remote sensing estimates and the actual plant heights. R^2^ (Equation (3)) measures the proportion of the variance in the actual plant heights that is explained by the remote sensing estimates. Furthermore, a Wilcoxon–Mann–Whitney test was conducted to determine if the differences between the RMSE and MAE values of plant height at different flight configurations were statistically significant.

(1) $$RMSE=\sqrt{\frac{\sum_{i=1}^{n}({H_{pred,i}-H_{actual,i})}^{2}}{n}}$$

(2) $$MAE=\frac{\sum_{i=1}^{n}{|H}_{pred,i}-H_{actual,\;i}|}{n}$$

(3) $$R^{2}=1-\frac{\sum_{i=1}^{n}({H_{pred,i}-H_{actual,i})}^{2}}{\sum_{i=1}^{n}({H_{pred,i}-{\bar{H}}_{actual,i})}^{2}}$$

where n is the number of plots, and ${\bar{H}}_{actual,i}$ is the mean of the actual plant heights. $H_{pred}$ denotes the predicted plant height estimated from remote sensing data, and $H_{actual}$ the actual plant height obtained from ground truth measurements.

#### 2.4.3. Sensor Fusion

The DTM generated from the LiDAR was used as the base layer to assess the effect of sensor fusion (plot-level fusion) on the accuracy of plant height estimation, which was subtracted from the DSM layers created with data from the RGB and multispectral sensors. The LiDAR’s DTM was chosen as the base because some studies [34,35] have indicated that LiDAR provides highly accurate and detailed topographical information, providing a reliable reference for ground elevation. Based on the values of R^2^, RMSE, and MAE, the results of sensor fusion were compared with the results of each specific sensor. The Wilcoxon–Mann–Whitney test was used to assess if there was any statistically significant difference (*p*-value < 0.05) between the evaluation metrics before and after sensor fusion.

## 3. Results and Discussion

### 3.1. Selecting the Best Height Metric

**Figure 2:**
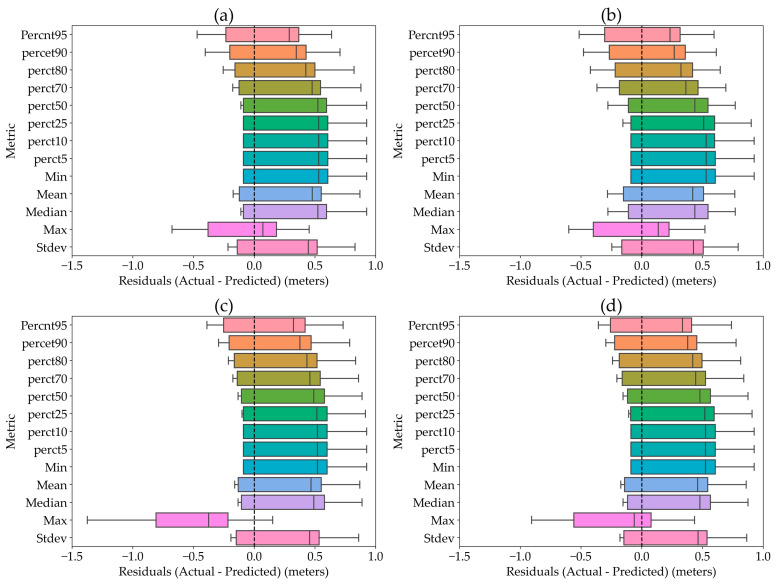
The residual (actual-predicted) values of plant height in dry peas estimation using data from LiDAR (**a**), Micasense MX (**b**), Zenmuse P1 (**c**), and Sentera 65r (**d**) sensors.

The results of the Wilcoxon–Mann–Whitney test showed that there was a difference (*p*-value = 0.0035) between the residual (actual-predicted) values of the maximum plant height and those of other plant height metrics, including minimum, mean, median, standard deviation, and percentiles, across all flight configurations and all sensors. This indicates that when measuring plant height in the dry pea using remote sensing sensors, the maximum estimated values should be prioritized for better accuracy. These findings are in agreement with previous studies [36,37] related to crop height measurement. This finding may be because, in short crops, like dry peas, the maximum plant height metric captures the full extent of growth potential more effectively than other metrics. The mean and median provide central tendency measures but may be less responsive to the extremes of plant growth. While standard deviation and percentiles offer insights into the variability and distribution of plant height, these metrics alone may not fully capture extreme values or outliers that significantly influence overall crop assessment. In addition, for all flight configurations, the average residual values for maximum plant height for both the LiDAR and Sentera 65r sensors were closer to the zero line, indicating more reliable plant height estimations compared to other sensors (Micasense and Zenmuse P1). The significant difference in residuals suggests that maximum height measurements are less susceptible to variations in flight altitude, flight speed, and image overlap, making that metric more reliable. The improved accuracy observed for maximum plant height estimations using LiDAR and Sentera 65r sensors could be attributed to their marginally wider field of view (FoV) and shorter focal lengths, which provide greater viewpoint separation and thus facilitate a more precise reconstruction of the 3D dense point cloud. These results align with the findings from the literature [37]. Figure 2 shows an example of a residual plot for a flight conducted at 46 m above ground level, with a speed of 10 m per second, and a forward and side overlap of 75% for different sensors.

Residual plots (Figure 2) show that the variability in height values is similar across sensors. Although LiDAR is expected to yield more accurate results at low altitudes [38], the observed similarity in variability among sensors can be attributed to several factors. First, we flew all sensors under identical flight conditions, minimizing effects from differences in altitude, speed, and overlap. Averaging data at the plot level likely reduced sensor-specific variability, especially for short crops like the dry pea. In addition, pre-processing steps such as outlier removal applied to all datasets would have standardized the resulting data. Lastly, external plant movement (e.g., wind) may have introduced a consistent level of variability across all sensors. However, LiDAR and Sentera 65r produced estimates more consistent with actual plant height.

### 3.2. The Effect of Flight Altitude on the Estimation of Plant Height

**Figure 3:**
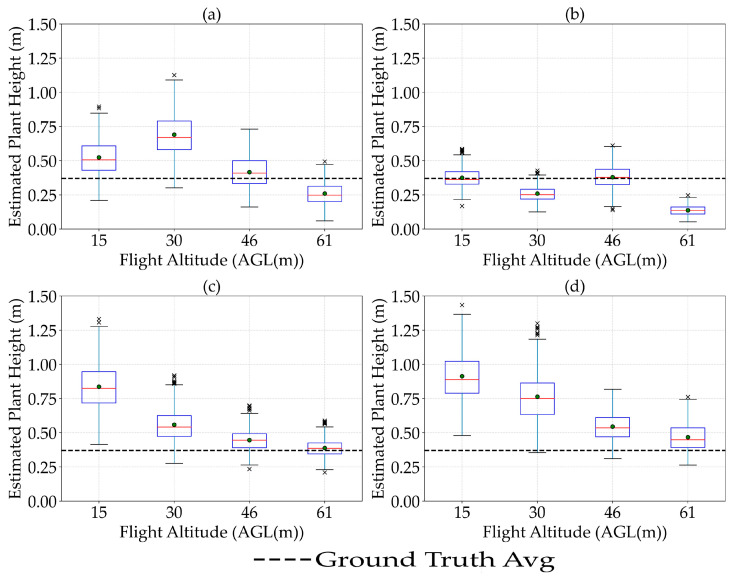
Effect of flight altitude on the estimation of plant height in dry pea when using LiDAR (**a**), Micasense MX (**b**), Zenmuse P1 (**c**), and Sentera 65r (**d**) sensors. × represents the outliers.

By increasing the flight altitude, the average value of plant height estimated by remote sensing sensors decreased. This reduction was especially pronounced for the RGB sensors (Zenmuse P1 and Sentera 65r), while the LiDAR and Micasense MX sensors exhibited more stable measurements across altitudes. To explore this further, plant height variability across flight altitudes is represented using box plots for all sensors, better reflecting the non-normal distribution of the data (Figure 3). These plots display the median, interquartile range (IQR), and outliers, providing a more straightforward representation of the distribution. The box plots show that both the mean plant height and IQR tend to decline with increasing altitude, with RGB sensors demonstrating the sharpest drop in both metrics. These results indicate that although measurements appear less variable at higher altitudes, they also become more biased, tending to underestimate plant height. In contrast, LiDAR data maintained a relatively consistent IQR and exhibited minimal sensitivity to altitude, indicating greater robustness and resilience to altitude-induced uncertainties in plant height estimation. This analysis was conducted to compare how different sensor types respond to altitude-induced resolution changes. Although GSD effects are known, the degree of sensitivity varies by sensor and requires quantification. The results highlight how higher altitudes introduce both reduced variability and increased bias, especially for RGB sensors. LiDAR and multispectral sensors showed greater resilience to altitude, offering practical insights for flight planning. This analysis helps identify the trade-off between measurement precision and accuracy across altitudes. Understanding altitude effects across sensors supports optimal data acquisition strategies in UAS-based phenotyping.

**Figure 4:**
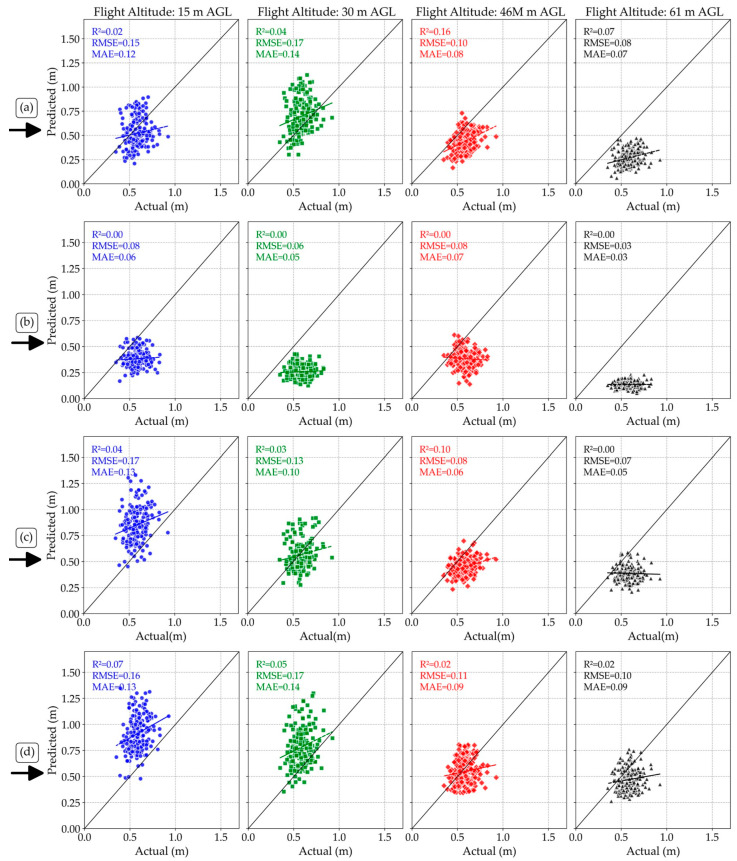
Comparisons of the estimated and the actual values of plant height under different flight altitudes for LiDAR (**a**), Micasense MX (**b**), Zenmuse P1 (**c**), and Sentera 65r (**d**) sensors. Actual—Actual plant height; m—meters.

A comparison of the estimated values of plant height under different flight altitudes for different sensors with the actual values (Figure 4) showed that increasing flight altitude consistently led to an underestimation of plant height in dry pea for all sensors tested. However, it should be emphasized that none of the sensors demonstrated strong predictive accuracy, as indicated by generally low R^2^ values. This underestimation was notably more pronounced for RGB sensors (Zenmuse P1 and Sentera 65r) compared to LiDAR and Micasense MX sensors. Additionally, Micasense MX consistently showed extreme underestimation across all flight altitudes compared to other sensors, possibly due to its lower ground resolution at higher altitudes, limiting its ability to accurately capture fine structural details of the plant canopy. Despite these observations, given the poor overall performance across all flight heights, these sensors may not be suitable for the reliable estimation of dry pea plant height in practical applications. These results show that predicted and actual plant heights are weakly correlated. This suggests the sensors struggle to accurately capture height across altitudes, likely due to multiple contributing factors.

This weak correlation can be attributed to the complex structure of the dry pea canopy, which likely hinders accurate 3D reconstruction by passive sensors. Photogrammetry methods (e.g., Structure from Motion) struggle to accurately resolve vertical structures in short, dense, overlapping foliage, particularly when images are captured at high altitudes, resulting in increasing ground sample distances (GSD) and lower resolution. LiDAR is an active remote sensing that captures detailed information about canopy structural variations, but its ability to detect small canopy variations, e.g., dry peas, is limited at higher altitudes due to reduced point density and larger footprint size.

These results align with previous studies that demonstrated the importance of canopy complexity and sensor resolution in influencing measurement error [39,40]. Furthermore, environmental factors such as variable illumination or wind-induced plant motion during flight missions may have contributed to measurement noise. LiDAR is generally more robust to such conditions, whereas passive sensors, such as RGB and multispectral systems, are more sensitive, as reported by [41] in studies estimating soybean height.

The results of the Wilcoxon–Mann–Whitney test showed no significant difference (*p*-value = 0.35) in residual errors (MAE and RMSE) across flight altitudes for any of the sensors. Although Figure 3 visually suggests a general trend of decreasing estimated plant height with increased flight altitude, the statistical analysis indicates these variations were not statistically significant. Thus, flying at higher altitudes could be considered a viable approach to reduce the cost and duration of flights without significantly compromising accuracy in estimating plant height for dry peas. However, the results of the Wilcoxon–Mann–Whitney test showed that there was a significant difference between the accuracy of the LiDAR sensor at a flight altitude of 46 m and the Sentera 65r sensor at 15 m versus the Micasense sensor at 61 m (*p*-value = 0.0012). This significant difference highlights the superiority of the LiDAR sensor at medium altitudes and the reduced accuracy of Micasense MX at higher altitudes.

**Figure 5:**
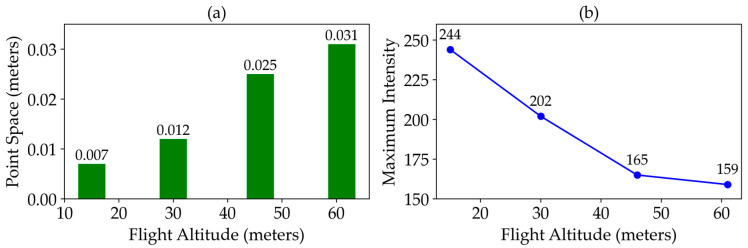
Effect of flight altitude on point distance (**a**) and maximum intensity (**b**) of LiDAR.

Increasing the platform altitude for UAS-based LiDAR data collection has several effects on the accuracy and completeness of the recorded data. Higher altitudes result in a lower frequency of returns per crown, leading to significant underestimation of individual crown areas and volumes. This is due to progressively fewer discrete first and last return combinations being recorded as the platform altitude increases [36], which impacts the distance, density, and detail of the point cloud data (Figure 5a). These results align with the findings of [42] that proved that increasing altitude may reduce RMSE values for marsh height estimation and in some cases, even slightly reduced RMSE values due to smoother representation of canopy variability at higher altitudes. Additionally, greater platform altitudes and larger footprint sizes reduce the intensity of the laser beam incident on surfaces (Figure 5b), thereby decreasing the probability of recording last returns above the noise threshold [36].

**Figure 6:**
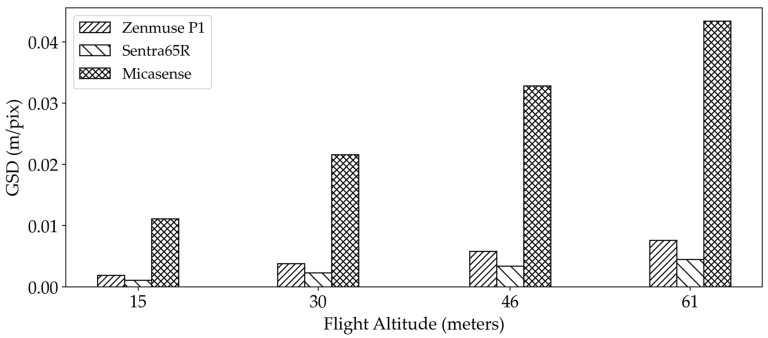
The sensitivity of the ground sampling distance (GSD) of different sensors to flight altitude.

The greater sensitivity of the RGB sensors to altitude changes could be attributed to sensor resolution and data accuracy. This is likely due to a higher ground sampling distance (GSD) at higher altitudes, which results in lower-resolution images (Figure 6). The coarser resolution can lead to less accurate height estimations as fine details and small variations in plant height become less distinguishable [43]. The decrease in resolution can increase the likelihood of underestimating the plant height, particularly in shorter crops like dry pea and for the Micasense sensor (lower resolution sensor among the sensors). This observation aligns with findings from other studies, where structure from motion techniques, often used with RGB sensors, were reported to lack the ability to accurately reconstruct the top of the canopy due to coarser spatial resolution and limited penetration capacity [37,44].

Ground sampling distance (GSD) (Equation (4)) increases linearly with flight altitude and inversely with the camera’s focal length and sensor resolution [45].

(4) $$GSD=\frac{H\cdot S}{f\;.\;I}$$

where *H* is the flight altitude above ground level (AGL), *S* is the sensor pixel size (in mm), *f* is the focal length (in mm), and *I* is the image width in pixels. As altitude increases, GSD also increases, resulting in fewer pixels per unit ground area. This reduction in spatial resolution causes fine canopy structures—especially the uppermost portions critical for accurate plant height measurements—to be poorly defined or completely missed during 3D reconstruction. For LiDAR, although point density also decreases with increasing altitude, it remains more robust due to its active sensing mechanism and direct distance measurements [45]. In contrast, RGB and multispectral sensors rely on image texture and structure-from-motion algorithms, which become increasingly ineffective with coarser GSD. These limitations are especially evident in shorter crops like dry peas, where subtle variations in canopy height are critical.

While RGB sensors like Zenmuse P1 and Sentera 65r showed greater sensitivity to altitude changes due to changes in image resolution, the LiDAR sensor maintained more stable height estimations across flight altitudes. This suggests that LiDAR is better suited for applications requiring precise plant height measurements across varying altitudes. In summary, while increasing flight altitude led to an underestimation of plant height for all sensors, the effect was more pronounced for RGB sensors. LiDAR remained the most accurate sensor at varying altitudes, followed by Zenmuse P1.

Despite variability across sensors and flight altitudes, the findings provide actionable guidance for UAS-based plant height monitoring in pulse crops. Specifically, for short-stature crops like dry peas, RGB sensors may not be suitable for precise height estimation, especially at higher altitudes, due to increased underestimation. In contrast, LiDAR demonstrates more consistent performance and can be used reliably across various altitudes. For resource-limited operations, low-altitude flights using high-resolution RGB sensors (e.g., Sentera 65r) may offer a cost-effective alternative when LiDAR is unavailable. Overall, the study supports sensor-specific and altitude-optimized flight planning to balance accuracy, cost, and operational efficiency.

### 3.3. The Effect of Flight Speed on the Estimation of Plant Height

**Figure 7:**
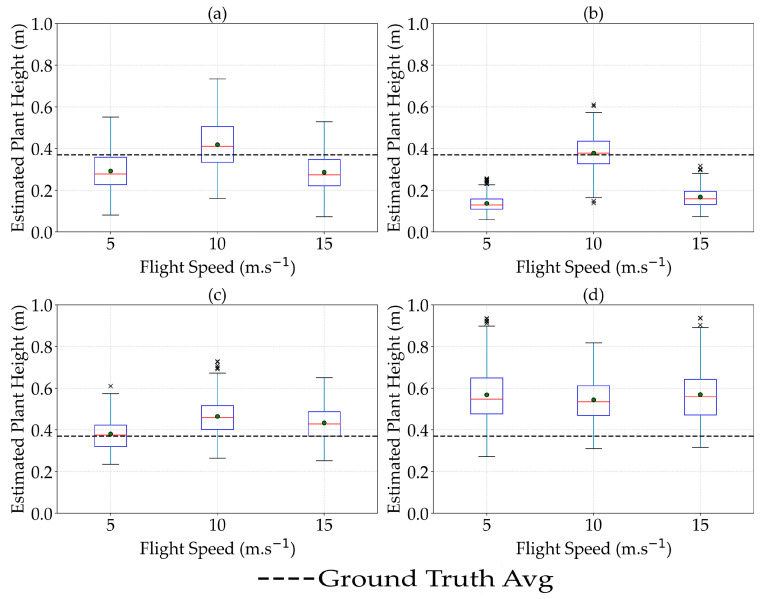
Effect of flight speed on the estimation of plant height in dry pea using LiDAR (**a**), Micasense MX (**b**), Zenmuse P1 (**c**), and Sentera 65r (**d**) sensors. × represents the outliers.

The plant height estimated by remote sensing sensors showed a fluctuating trend at different flight speeds, especially for LiDAR and multispectral sensors compared to RGB sensors (Figure 7). Overall, these results suggest sensor-dependent sensitivity to changes in flight speed, with LiDAR showing the most pronounced response. However, it is important to emphasize that while LiDAR exhibited clear changes, the variation for Micasense MX was comparatively moderate rather than sharp.

It is critical to acknowledge that the initial differences among sensors in the estimated plant height (ranging from approximately 0.15 to 0.58 m) indicate substantial discrepancies when compared to actual measured values, raising questions about the overall accuracy and utility of these sensors for precise plant height estimation in dry peas. Thus, even though flight speed influences the estimated heights to some degree, the primary concern remains the fundamental accuracy of the sensor-derived measurements.

While numerous studies have highlighted the adverse effects of higher flight speeds on the accuracy of plant height estimation using photogrammetric and LiDAR-based methods [46,47], our findings indicated no statistically significant differences in RMSE and MAE values across different flight speeds for all sensors when estimating plant height in dry peas (Appendix A-Figure A1). This discrepancy could be attributed to the specific characteristics of dry pea fields in North Dakota, which are relatively homogeneous and flat, thereby mitigating the impact of flight speed on data accuracy. The high quality of sensors used, such as the Zenmuse P1 and Micasense MX, with advanced image stabilization and fast shutter speeds, and our optimized flight planning ensured sufficient image overlap and minimized data gaps, even at higher speeds. However, our analysis of R^2^ values suggested that optimal flight speeds differed across sensors, with 10 m.s^−1^ performing better for LiDAR, Zenmuse P1, and Micasense MX sensors, while 5 m.s^−1^ was preferable for the Sentera 65r sensor. These findings underscore the need for tailored flight parameters based on specific sensor capabilities and field conditions. In conclusion, while our study suggested that flight speed may have a limited impact on the accuracy of plant height estimation in dry peas, these findings may not be universally applicable. Future research should explore the effects of flight speed in more complex terrains and with different crop types to better understand the nuances of UAV-based data collection in agriculture.

### 3.4. The Effect of Image Overlaps on Plant Height Estimation

**Figure 8:**
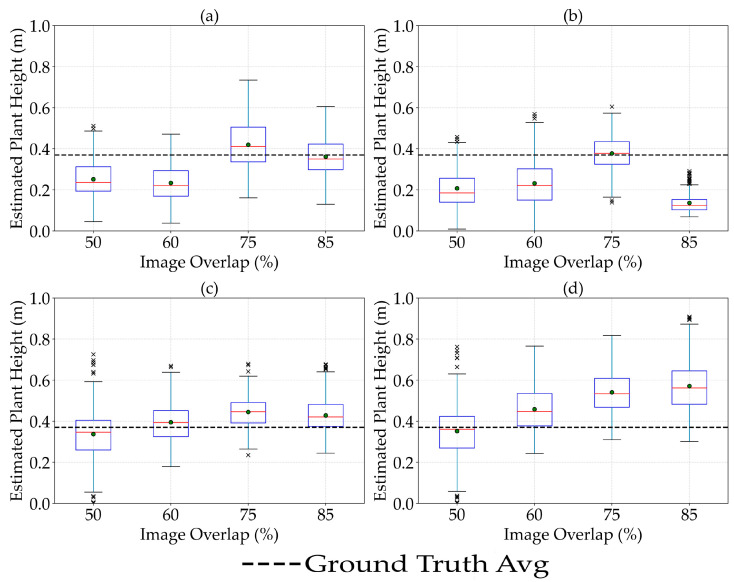
Effect of image overlap on the estimation of plant height in dry pea at LiDAR (**a**), Micasense MX (**b**), Zenmuse P1 (**c**), and Sentra 65R (**d**) sensors. × represents the outliers.

The effect of image overlap (side and forward) on the plant height estimation in dry pea depended on the type of sensors used. The estimation of plant height using LiDAR (Figure 8a) demonstrated sensitivity to image overlap, highlighting inconsistent trends with changes in overlap percentage. Lower overlaps initially led to underestimation, whereas higher overlaps (particularly at 75%) improved the accuracy, suggesting optimal overlap configurations might exist to enhance plant height estimation. Similarly, the Micasense sensor also showed improved estimation at 75% overlap, with a slight decline beyond this threshold (Figure 8b). These findings imply that excessively high overlap may not always yield improvements in accuracy and that an intermediate overlap (around 75%) could be optimal for capturing sufficient canopy details without excessive data redundancy. Regarding RGB sensors, including Zenmuse P1 (Figure 8c) and Sentera 65r (Figure 8d), increasing the image overlap sharply increased the average estimated plant height, with the highest value at 85% overlap. The rate of increase in the estimated value of plant height for Sentera 65r was greater than that for the Zenmuse P1 sensor.

Comparing the actual values vs. estimated values of plant height in dry peas at different image overlap values (Appendix B-Figure A2) showed that increasing the image overlap improved the accuracy of plant height estimation in all UAS sensors. Although the Wilcoxon–Mann–Whitney test showed no significant differences (*p* < 0.05) among the RMSE and MAE values of plant height at different image overlap settings, the R^2^ value showed an improvement at higher image overlap values, especially for the LiDAR and RGB sensors. The highest level of overlap, specifically 75%, reduced the amount of underestimation of plant height for all sensors, particularly for the Sentera 65r. This suggests that while users can set the image overlap to lower values (50% or 60%) to reduce flight time during data collection, an image overlap of 75% may result in better accuracy when estimating plant height for dry peas, especially with the Sentera 65r sensor.

Image overlap can affect the accuracy of plant height estimation by influencing measurement techniques, data processing, and algorithm performance. Higher overlap improves the quality of Micasense MX data by reducing shadow effects and enhancing the signal-to-noise ratio, thereby improving height estimation. RGB sensors benefit significantly from increased overlap as it improves image alignment and stitching, leading to better 3D reconstruction and height estimation. The increase in height estimation with increased overlap indicates improved data consistency and reduced errors in photogrammetry processes. Higher overlap provides more data points for algorithms to process, leading to better model accuracy. For LiDAR, the fluctuating trend may result from the algorithm’s sensitivity to point density variations. In contrast, photogrammetric algorithms for RGB sensors benefit more linearly from increased overlap due to improved image matching and feature extraction [37].

### 3.5. Effect of Sensor Fusion on Plant Height Estimation Accuracy

**Figure 9:**
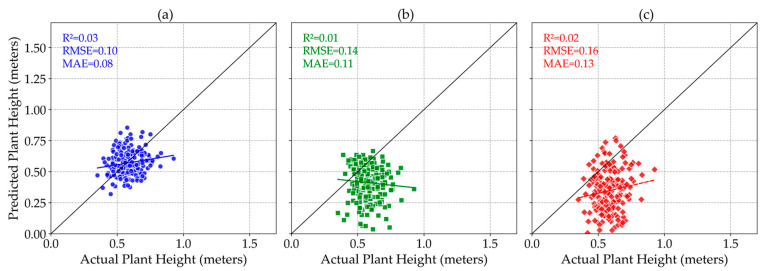
Effect of sensor fusion on the accuracy of plant height estimation of Zenmuse P1 (**a**), Sentera 65r (**b**), and Micasense MX (**c**) sensors.

Although using the LiDAR’s DTM layer as a reference for plant height estimation only improved the *R*^2^ value for the Zenmuse P1 sensor, while decreasing the accuracy for both the Sentera 65r and Micasense MX sensors, all sensors still exhibit poor performance in accurately estimating plant height in dry peas, as evidenced by low *R*^2^ values and varying error metrics (Figure 9). The results of the Wilcoxon–Mann–Whitney test showed that the difference between using sensor fusion and estimating plant height with individual sensors was not statistically significant for any of the sensors.

### 3.6. Limitations of Sensor Performance for Estimating Plant Height

Although sensor comparisons revealed differences in performance metrics (R^2^, RMSE, MAE), overall model fits were weak across all tested sensors, limiting their practical reliability for accurately estimating plant height in dry peas. The LiDAR sensor provided the best relative performance, indicated by the highest R^2^ (0.16), lowest RMSE (0.10), and lowest MAE (0.08). The Zenmuse P1 sensor followed with a modest R^2^ (0.10), along with relatively low error metrics (RMSE = 0.08 and MAE = 0.08). Notably, while the Micasense sensor exhibited low RMSE and MAE values, it had an R^2^ of 0, meaning it failed to explain variability in plant height, highlighting significant limitations for practical use. The Sentera 65r sensor similarly demonstrated limited accuracy. Overall, none of the sensors demonstrated strong predictive accuracy for plant height in dry peas under the flight conditions tested, indicating the need for caution when applying these sensors for precise plant height estimation in practical scenarios.

**Figure 10:**
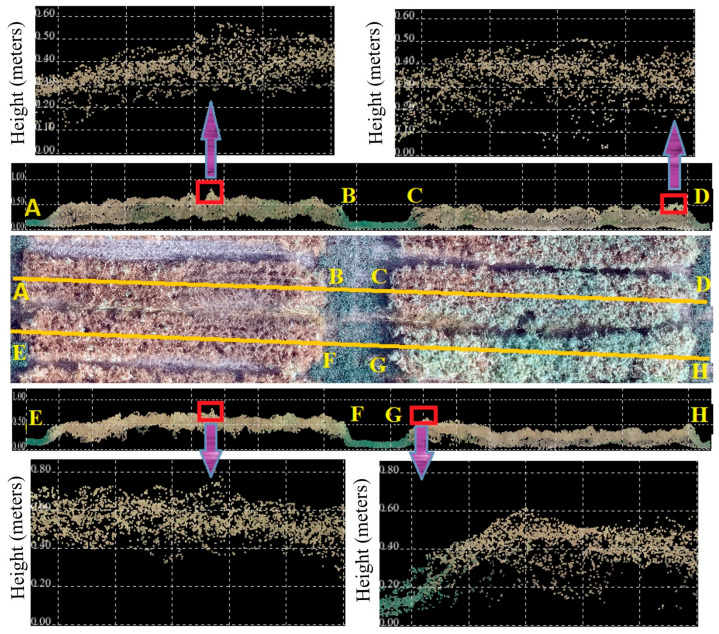
Effect of the canopy’s structure and growth pattern on the accuracy of plant height using LiDAR. AB and EF profiles show plots with disparate structure, while CD and GH profiles show plots with dense structure.

Although LiDAR demonstrated the best accuracy for estimating the plant height of dry pea at different flight altitudes compared to RGB and multispectral sensors, its results were still not sufficiently accurate and reliable. Several factors contribute to this inaccuracy. Firstly, the structure and growth patterns of crops significantly affect the accuracy of plant height estimation using remote sensing technologies. Short and dense crops such as dry pea, with their planophile leaf angles and complex canopy structures, present more challenges compared to tall and sparse crops (Figure 10). The dense and uneven canopies of dry pea can hinder the penetration and return of laser pulses in LiDAR measurements, reducing accuracy [37]. Similarly, the spatial resolution of imagery from photogrammetry can fail to capture fine variations in canopy height, further contributing to measurement errors. Therefore, understanding the specific structural characteristics of the crop canopy is crucial for improving the accuracy of plant height estimations using remote sensing methods. These findings align with the results of previous studies which highlight the difficulty of measuring plant height using LiDAR in cotton [47], potato and sugar beet [13].

Moreover, the design of the LiDAR sensor affects the accuracy of ground height estimates, particularly discrete return systems like the one used in the study, which struggle to discern multiple returns less than two meters high [42] due to short time intervals between subsequent returns from the same laser pulse. Consequently, in environments with dense vegetative cover under two meters in canopy height, subsequent ground returns might not be recorded, leading to inaccuracies in ground height estimation. Therefore, it is recommended to test other LiDAR sensors and compare their results with these findings in future studies.

### 3.7. Challenges and Future Works

The reasons for the deviations in height estimates using different sensors can be caused by technical specifications of the sensors and the crop target used on this study. The Micasense MX has been created for multispectral imaging and may not deliver sufficient spatial resolution for precise height measurements. As mentioned above, LiDAR sensor might struggle to perform well with canopies with less than two meters high, which might have contributed to the noisy data, affecting the accuracy of the crop height measurements. As for the high resolution RGB sensors (Zenmuse P1 and Sentera 65r), they rely on photogrammetry techniques to capture heigh information, which can be influenced by changing lighting, plant density, and overlapping leaves.

The dense canopy structure of dry pea, with overlapping leaves and complex stem arrangements, poses challenges for both RGB cameras and LiDAR in accurately estimating overall plant height at the plot level. Other environmental conditions like lighting changes, and wind can also make the RGB and LiDAR measurements less accurate. Vegetation characteristics, such as species, canopy height and orientation, stem density, canopy cover, and spectral properties, have been documented as important contributing factors to errors in LiDAR ground height estimates in marshes and other environments [42]. The lower accuracy of RGB and multispectral sensors compared to LiDAR in estimating plant height in dry pea can be attributed to the challenges SfM faces in accurately reconstructing the top of the canopy. As discussed in the study by [37], one reason for this discrepancy is the difference in spatial resolution between LiDAR and the RGB and multispectral cameras used in SfM, relative to the size of objects at the top of the canopy. However, increasing the spatial resolution in SfM can lead to noisier dense clouds with more gaps over vegetated areas.

Additionally, the impact of image resolution on the accuracy of height estimation needs further investigation. While higher resolution images capture more canopy details, they may not necessarily improve accuracy, as they can introduce additional noise in dense vegetation. Conversely, lower-resolution images may smooth the canopy surface but fail to capture fine-scale height variations. Future studies should systematically assess the effect of resolution on height estimation for different sensor types. Another consideration is the use of ground control points (GCPs). This study did not incorporate GCPs, which could introduce elevation inconsistencies across different flights. Future work should evaluate the extent to which GCPs improve the consistency of elevation data across multiple flight configurations. In field measurements, an important factor influencing underestimation could be the methodology used for ground-truthing. If crew members had to lift plants off the ground to measure plant height, this could partially explain discrepancies between measured and estimated heights. Investigating the impact of manual plant height measurements on validation accuracy would be beneficial for refining future methodologies. Moreover, the presence of tendrils in dry pea canopies may affect the quality of image stitching in SfM. These fine structures, which extend above the main canopy, create challenges for the algorithm when aligning overlapping images, potentially contributing to reconstruction errors. Future research should explore preprocessing techniques or alternative modeling approaches to mitigate this issue.

Finally, different data processing and analysis techniques could influence the accuracy of plant height estimation. Investigating alternative machine learning models, deep learning-based canopy height reconstruction, or hybrid approaches that integrate LiDAR and SfM-derived point clouds may offer improvements [48]. Future studies should explore these methodologies to enhance plant height estimation in dense, short-stature crops.

Future work should focus in exploring advanced algorithms to improve canopy reconstruction when using SfM when using high-resolution RGB and multispectral cameras, coupled with noise reduction techniques to enhance plant height estimation reliability. Furthermore, for LiDAR, the processing algorithms and their ability to process through dense canopies and separate ground and vegetation returns are also important. Ground truth data are crucial to validate sensor measurements, and any noise or variance in these data could lead to inconsistent results. Moreover, integrating multiple sensors with machine learning models can enhance accuracy. To achieve reliable results, high-quality ground truth data should be collected under consistent environmental conditions, supported by advanced data processing algorithms and calibration methods.

## 4. Conclusions

As a case study on the use of UAS-based remote sensing technologies for estimating plant height in short crops, this study has addressed existing gaps in the literature. This research has evaluated the performance of LiDAR, RGB, and multispectral sensors under various flight configurations and image overlaps. It also explores methods to improve the accuracy of plant height estimation. In summary, these results have indicated that while LiDAR is the most accurate sensor available to estimate the plant height in the dry pea, not all tested sensors have had a reliable performance in the present study. However, the outputs of the fusion approach differed little from those of the single sensors, indicating that collecting data from multiple sensors may not always guarantee a high level of precision. The findings have highlighted that different flight altitudes maintain accuracy for several sensors, allowing reduced operational time and cost. In the case of the RedEdge-MX sensor, though, accuracy was more sensitive to altitude changes, suggesting that lower flight altitudes are preferable for optimal performance. Furthermore, while multispectral and LiDAR sensors have been more affected by changes in flight speed, the overall accuracy, as indicated by RMSE and MAE values, remained consistent across varying speeds. This means that with well-tuned flight parameters, maximum efficiency can be obtained without compromising data quality. In summary, this study has addressed a gap in the literature by evaluating sensor performance across diverse flight configurations for low-lying crops. These results have underscored difficulties with canopies with complex structure and suggested great promise for merging machine learning models with LiDAR and other sensor data.

## Figures and Tables

**Figure A1:**
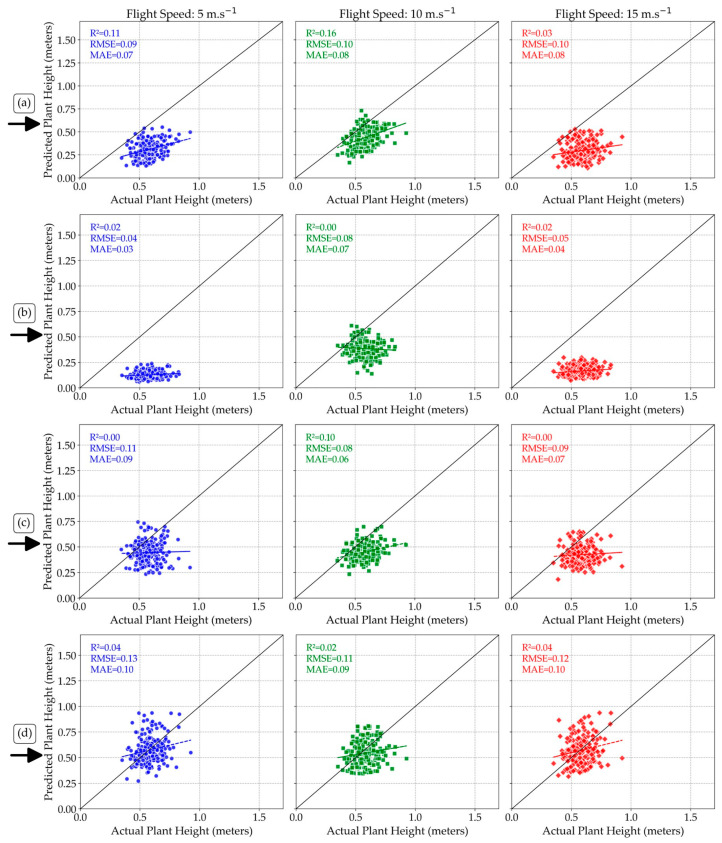
Comparing the estimated with the actual values of plant height under different flight speeds for LiDAR (**a**), Micasense MX (**b**), Zenmuse P1 (**c**), and Sentera 65r (**d**) sensors.

**Figure A2:**
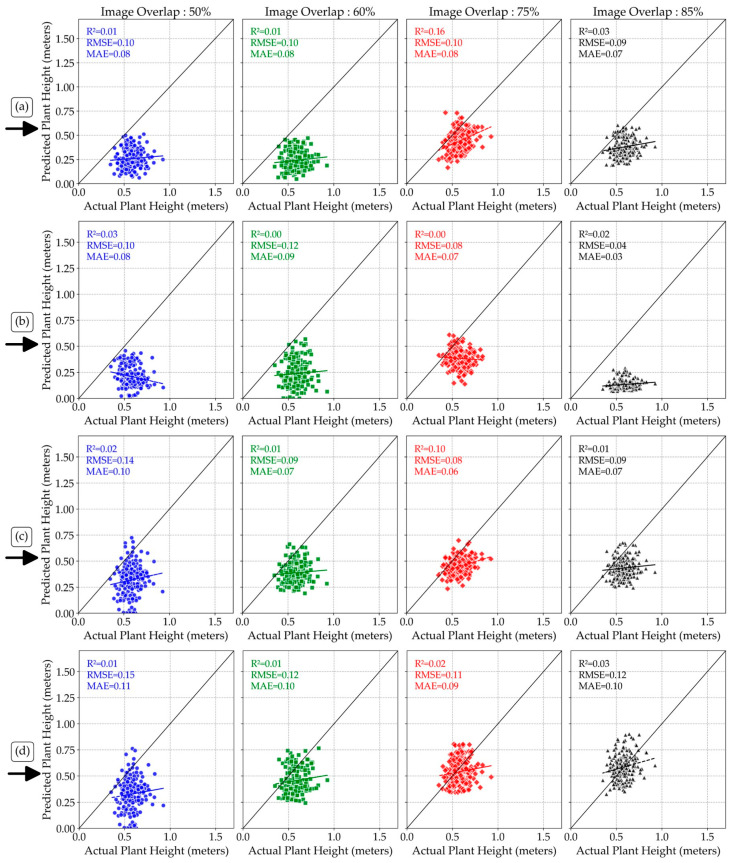
Comparing the estimated with the actual values of plant height under different image overlaps for LiDAR (**a**), Micasense MX (**b**), Zenmuse P1 (**c**), and Sentera 65r (**d**) sensors.

## Data Availability

Data will be available upon request.
